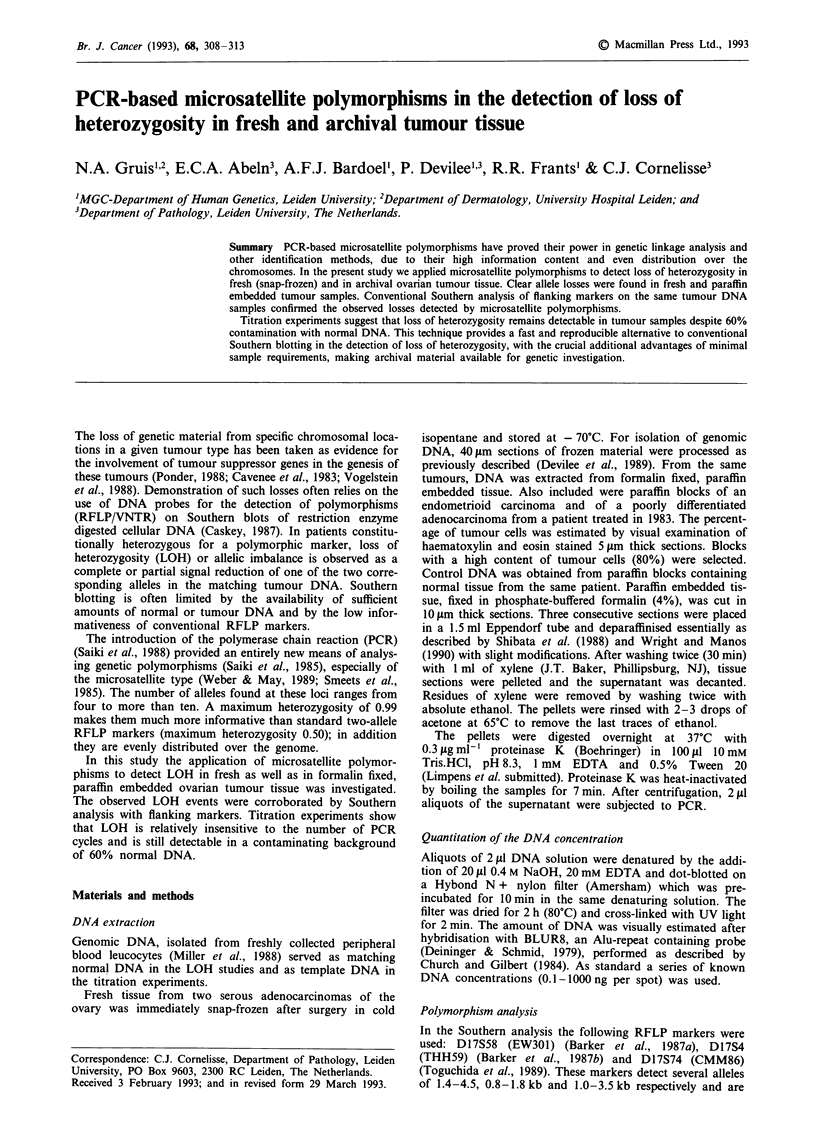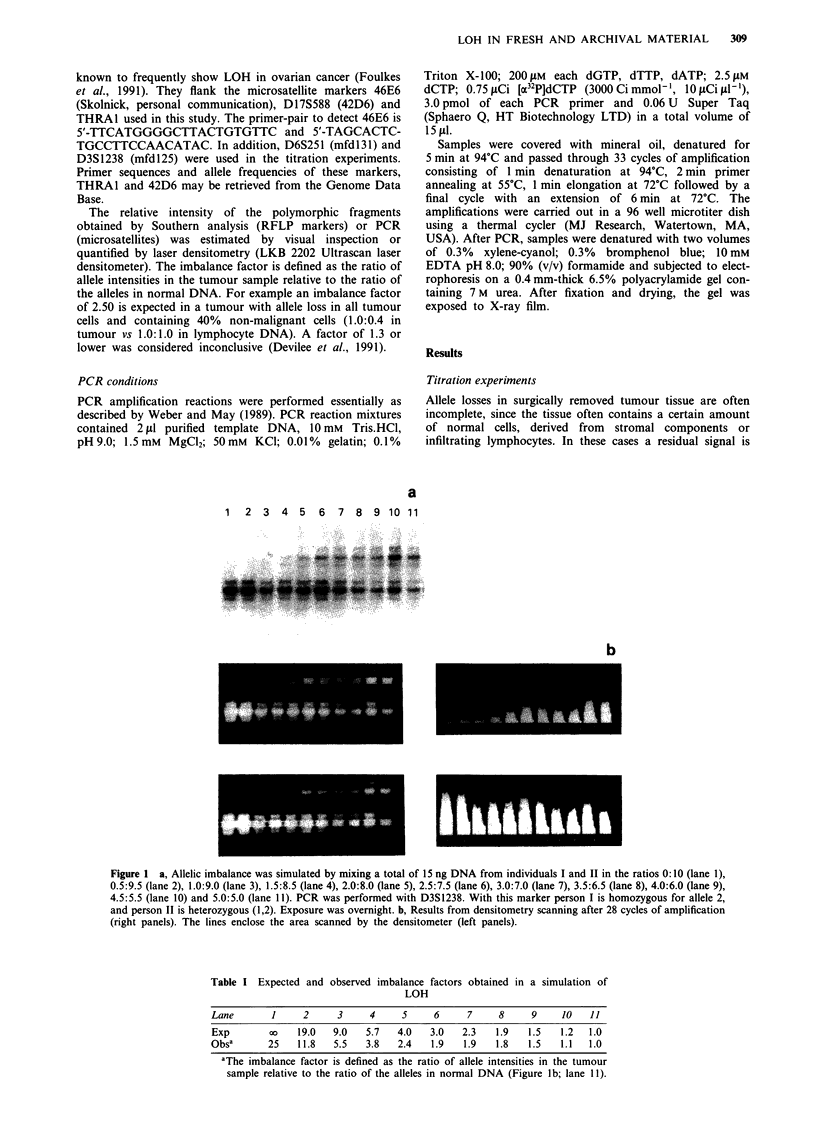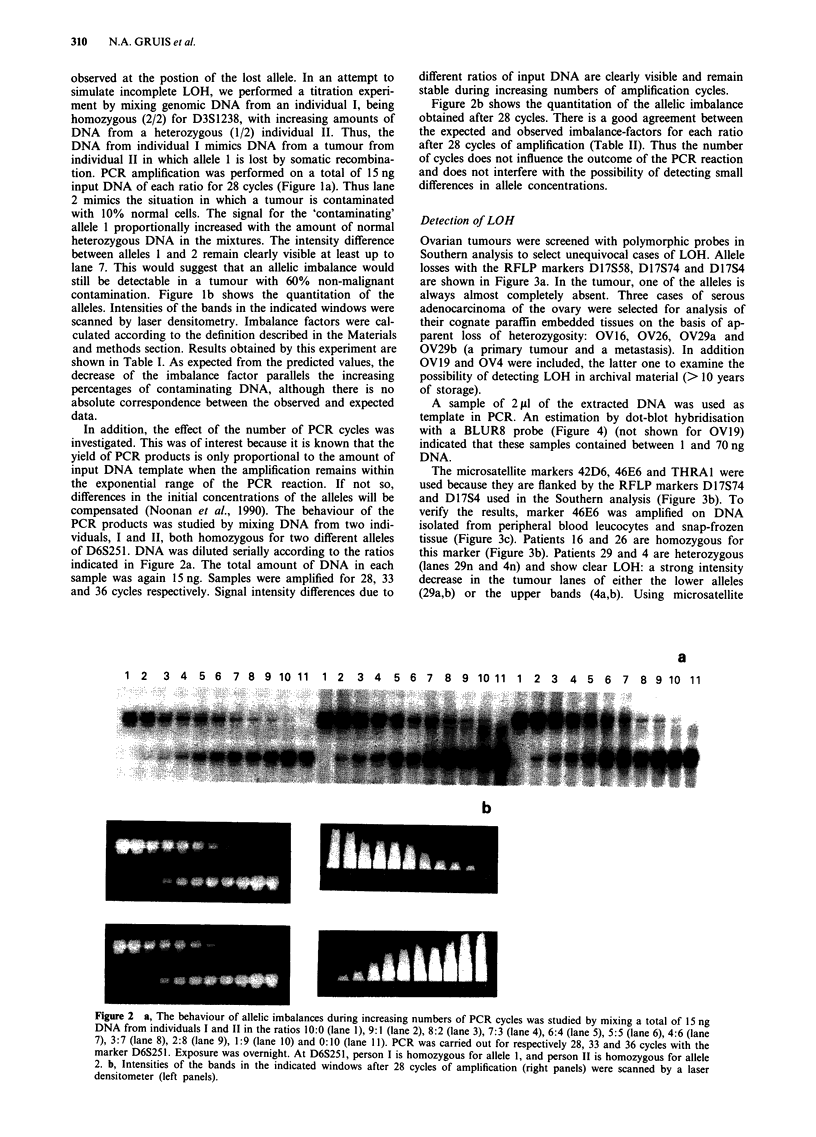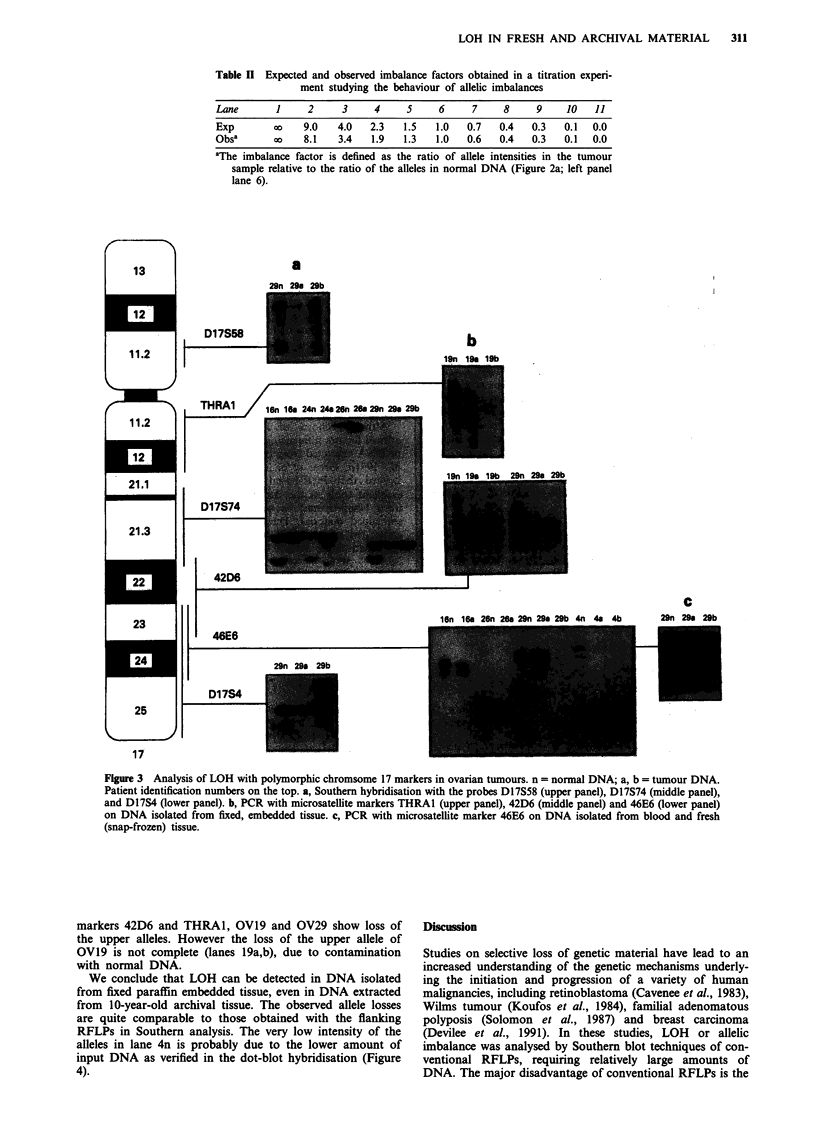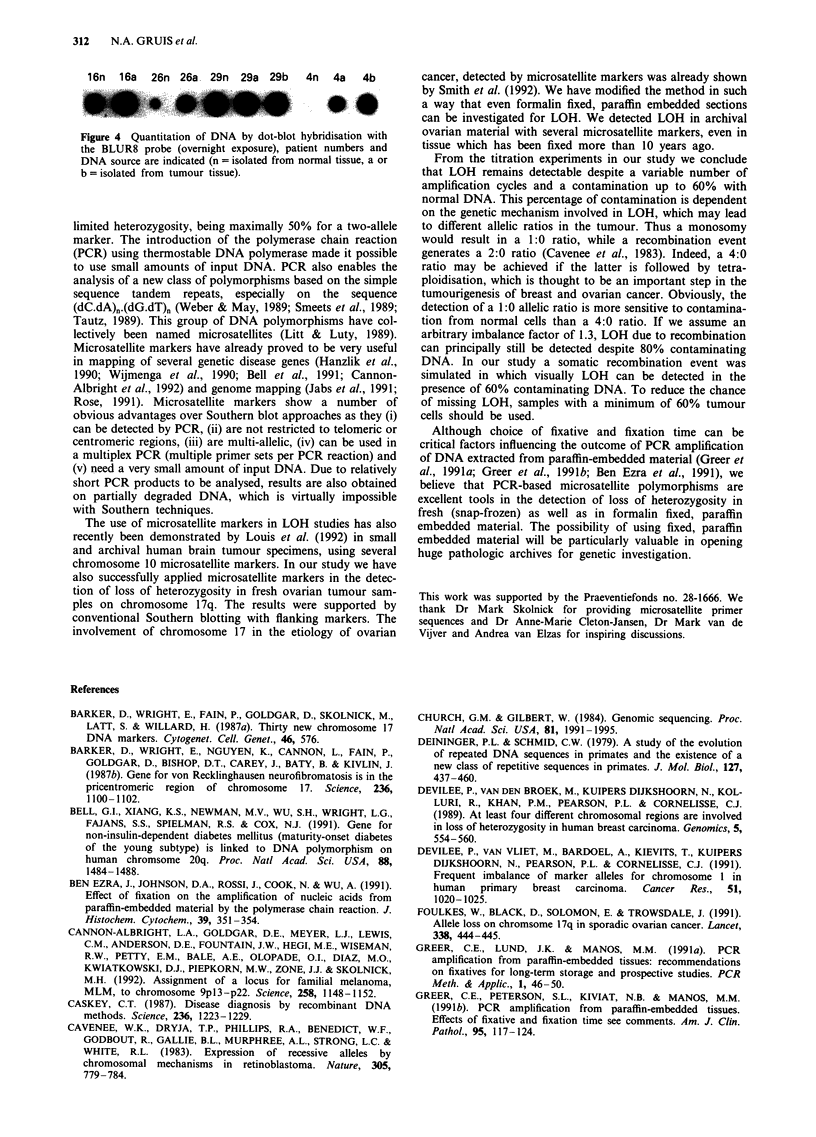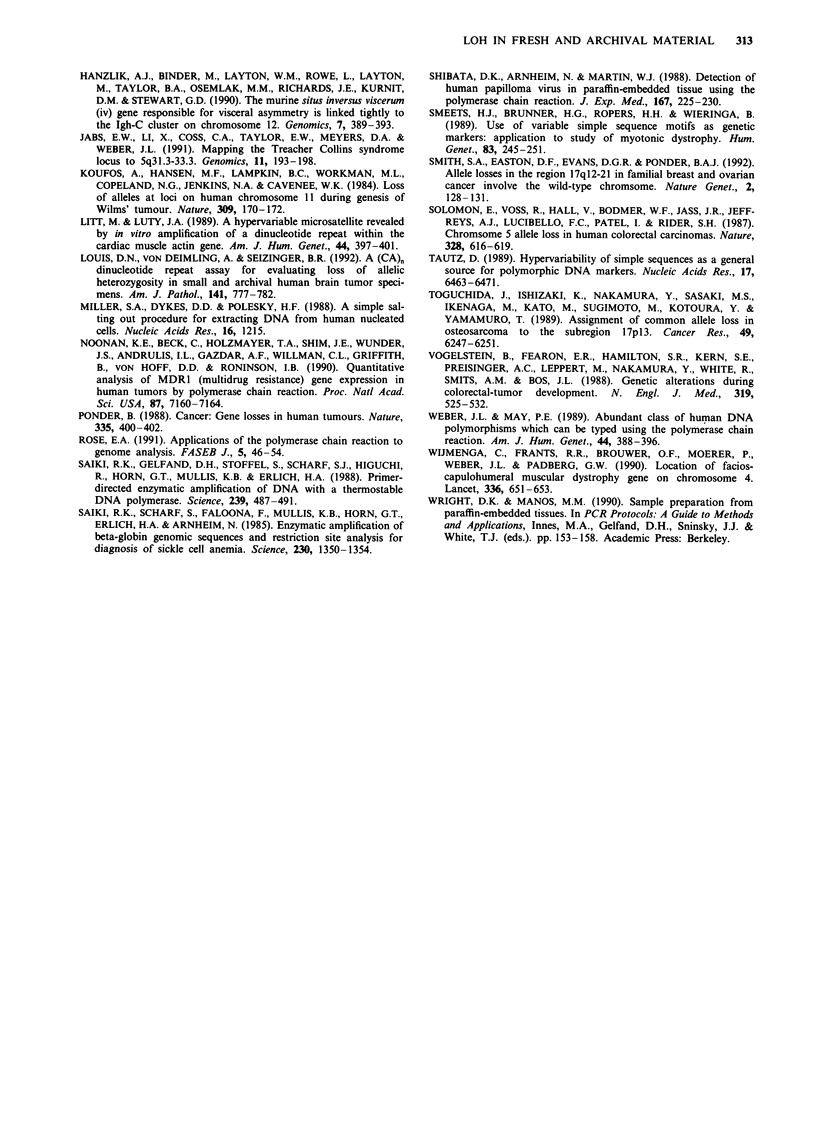# PCR-based microsatellite polymorphisms in the detection of loss of heterozygosity in fresh and archival tumour tissue.

**DOI:** 10.1038/bjc.1993.333

**Published:** 1993-08

**Authors:** N. A. Gruis, E. C. Abeln, A. F. Bardoel, P. Devilee, R. R. Frants, C. J. Cornelisse

**Affiliations:** MGC-Department of Human Genetics, Leiden University, The Netherlands.

## Abstract

**Images:**


*Br. J. Cancer* (1993), **68**, 308–313 © Macmillan Press Ltd., 1993

### PCR-based microsatellite polymorphisms in the detection of loss of heterozygosity in fresh and archival tumour tissue

N.A. Gruis[1,2], E.C.A. Abeln[3], A.F.J. Bardoel[1], P. Devilee[1,3], R.R. Frants[1] & C.J. Cornelisse[3]

[1]*MGC-Department of Human Genetics, Leiden University;* [2]*Department of Dermatology, University Hospital Leiden; and* [3]*Department of Pathology, Leiden University, The Netherlands.*

**Summary** PCR-based microsatellite polymorphisms have proved their power in genetic linkage analysis and other identification methods, due to their high information content and even distribution over the chromosomes. In the present study we applied microsatellite polymorphisms to detect loss of heterozygosity in fresh (snap-frozen) and in archival ovarian tumour tissue. Clear allele losses were found in fresh and paraffin embedded tumour samples. Conventional Southern analysis of flanking markers on the same tumour DNA samples confirmed the observed losses detected by microsatellite polymorphisms.

Titration experiments suggest that loss of heterozygosity remains detectable in tumour samples despite 60% contamination with normal DNA. This technique provides a fast and reproducible alternative to conventional Southern blotting in the detection of loss of heterozygosity, with the crucial additional advantages of minimal sample requirements, making archival material available for genetic investigation.

The loss of genetic material from specific chromosomal locations in a given tumour type has been taken as evidence for the involvement of tumour suppressor genes in the genesis of these tumours (Ponder, 1988; Cavenee *et al.*, 1983; Vogelstein *et al.*, 1988). Demonstration of such losses often relies on the use of DNA probes for the detection of polymorphisms (RFLP/VNTR) on Southern blots of restriction enzyme digested cellular DNA (Caskey, 1987). In patients constitutionally heterozygous for a polymorphic marker, loss of heterozygosity (LOH) or allelic imbalance is observed as a complete or partial signal reduction of one of the two corresponding alleles in the matching tumour DNA. Southern blotting is often limited by the availability of sufficient amounts of normal or tumour DNA and by the low informativeness of conventional RFLP markers.

The introduction of the polymerase chain reaction (PCR) (Saiki *et al.*, 1988) provided an entirely new means of analysing genetic polymorphisms (Saiki *et al.*, 1985), especially of the microsatellite type (Weber & May, 1989; Smeets *et al.*, 1985). The number of alleles found at these loci ranges from four to more than ten. A maximum heterozygosity of 0.99 makes them much more informative than standard two-allele RFLP markers (maximum heterozygosity 0.50); in addition they are evenly distributed over the genome.

In this study the application of microsatellite polymorphisms to detect LOH in fresh as well as in formalin fixed, paraffin embedded ovarian tumour tissue was investigated. The observed LOH events were corroborated by Southern analysis with flanking markers. Titration experiments show that LOH is relatively insensitive to the number of PCR cycles and is still detectable in a contaminating background of 60% normal DNA.

### Materials and methods

### *DNA extraction*

Genomic DNA, isolated from freshly collected peripheral blood leucocytes (Miller *et al.*, 1988) served as matching normal DNA in the LOH studies and as template DNA in the titration experiments.

Fresh tissue from two serous adenocarcinomas of the ovary was immediately snap-frozen after surgery in cold isopentane and stored at − 70°C. For isolation of genomic DNA, 40 μm sections of frozen material were processed as previously described (Devilee *et al.*, 1989). From the same tumours, DNA was extracted from formalin fixed, paraffin embedded tissue. Also included were paraffin blocks of an endometrioid carcinoma and of a poorly differentiated adenocarcinoma from a patient treated in 1983. The percentage of tumour cells was estimated by visual examination of haematoxylin and eosin stained 5 μm thick sections. Blocks with a high content of tumour cells (80%) were selected. Control DNA was obtained from paraffin blocks containing normal tissue from the same patient. Paraffin embedded tissue, fixed in phosphate-buffered formalin (4%), was cut in 10 μm thick sections. Three consecutive sections were placed in a 1.5 ml Eppendorf tube and deparaffinised essentially as described by Shibata *et al.* (1988) and Wright and Manos (1990) with slight modifications. After washing twice (30 min) with 1 ml of xylene (J.T. Baker, Phillipsburg, NJ), tissue sections were pelleted and the supernatant was decanted. Residues of xylene were removed by washing twice with absolute ethanol. The pellets were rinsed with 2–3 drops of acetone at 65°C to remove the last traces of ethanol.

The pellets were digested overnight at 37°C with 0.3 $\mu g\ ml^{-1}$ proteinase K (Boehringer) in 100 μl 10 mM Tris.HCl, pH 8.3, 1 mM EDTA and 0.5% Tween 20 (Limpens *et al.* submitted). Proteinase K was heat-inactivated by boiling the samples for 7 min. After centrifugation, 2 μl aliquots of the supernatant were subjected to PCR.

### *Quantitation of the DNA concentration*

Aliquots of 2 μl DNA solution were denatured by the addition of 20 μl 0.4 M NaOH, 20 mM EDTA and dot-blotted on a Hybond N+ nylon filter (Amersham) which was pre-incubated for 10 min in the same denaturing solution. The filter was dried for 2 h (80°C) and cross-linked with UV light for 2 min. The amount of DNA was visually estimated after hybridisation with BLUR8, an Alu-repeat containing probe (Deininger & Schmid, 1979), performed as described by Church and Gilbert (1984). As standard a series of known DNA concentrations (0.1–1000 ng per spot) was used.

### *Polymorphism analysis*

In the Southern analysis the following RFLP markers were used: D17S58 (EW301) (Barker *et al.*, 1987*a*), D17S4 (THH59) (Barker *et al.*, 1987*b*) and D17S74 (CMM86) (Toguchida *et al.*, 1989). These markers detect several alleles of 1.4–4.5, 0.8–1.8 kb and 1.0–3.5 kb respectively and are

Correspondence: C.J. Cornelisse, Department of Pathology, Leiden University, PO Box 9603, 2300 RC Leiden, The Netherlands.
Received 3 February 1993; and in revised form 29 March 1993.

known to frequently show LOH in ovarian cancer (Foulkes *et al.*, 1991). They flank the microsatellite markers 46E6 (Skolnick, personal communication), D17S588 (42D6) and THRA1 used in this study. The primer-pair to detect 46E6 is 5'-TTCATGGGGCTTACTGTGTTC and 5'-TAGCACTC-TGCCTTCCAACATAC. In addition, D6S251 (mfd131) and D3S1238 (mfd125) were used in the titration experiments. Primer sequences and allele frequencies of these markers, THRA1 and 42D6 may be retrieved from the Genome Data Base.

The relative intensity of the polymorphic fragments obtained by Southern analysis (RFLP markers) or PCR (microsatellites) was estimated by visual inspection or quantified by laser densitometry (LKB 2202 Ultrascan laser densitometer). The imbalance factor is defined as the ratio of allele intensities in the tumour sample relative to the ratio of the alleles in normal DNA. For example an imbalance factor of 2.50 is expected in a tumour with allele loss in all tumour cells and containing 40% non-malignant cells (1.0:0.4 in tumour *vs* 1.0:1.0 in lymphocyte DNA). A factor of 1.3 or lower was considered inconclusive (Devilee *et al.*, 1991).

### *PCR conditions*

PCR amplification reactions were performed essentially as described by Weber and May (1989). PCR reaction mixtures contained 2 µl purified template DNA, 10 mM Tris.HCl, pH 9.0; 1.5 mM $MgCl_2$; 50 mM KCl; 0.01% gelatin; 0.1% Triton X-100; 200 µM each dGTP, dTTP, dATP; 2.5 µM dCTP; 0.75 µCi [$\alpha^{32}$P]dCTP (3000 Ci $mmol^{-1}$, 10 µCi $\mu l^{-1}$), 3.0 pmol of each PCR primer and 0.06 U Super Taq (Sphaero Q, HT Biotechnology LTD) in a total volume of 15 µl.

Samples were covered with mineral oil, denatured for 5 min at 94°C and passed through 33 cycles of amplification consisting of 1 min denaturation at 94°C, 2 min primer annealing at 55°C, 1 min elongation at 72°C followed by a final cycle with an extension of 6 min at 72°C. The amplifications were carried out in a 96 well microtiter dish using a thermal cycler (MJ Research, Watertown, MA, USA). After PCR, samples were denatured with two volumes of 0.3% xylene-cyanol; 0.3% bromphenol blue; 10 mM EDTA pH 8.0; 90% (v/v) formamide and subjected to electrophoresis on a 0.4 mm-thick 6.5% polyacrylamide gel containing 7 M urea. After fixation and drying, the gel was exposed to X-ray film.

### Results

### *Titration experiments*

Allele losses in surgically removed tumour tissue are often incomplete, since the tissue often contains a certain amount of normal cells, derived from stromal components or infiltrating lymphocytes. In these cases a residual signal is

**Figure 1** **a**, Allelic imbalance was simulated by mixing a total of 15 ng DNA from individuals I and II in the ratios 0:10 (lane 1), 0.5:9.5 (lane 2), 1.0:9.0 (lane 3), 1.5:8.5 (lane 4), 2.0:8.0 (lane 5), 2.5:7.5 (lane 6), 3.0:7.0 (lane 7), 3.5:6.5 (lane 8), 4.0:6.0 (lane 9), 4.5:5.5 (lane 10) and 5.0:5.0 (lane 11). PCR was performed with D3S1238. With this marker person I is homozygous for allele 2, and person II is heterozygous (1,2). Exposure was overnight. **b**, Results from densitometry scanning after 28 cycles of amplification (right panels). The lines enclose the area scanned by the densitometer (left panels).

**Table I** Expected and observed imbalance factors obtained in a simulation of LOH

| *Lane* | *1* | *2* | *3* | *4* | *5* | *6* | *7* | *8* | *9* | *10* | *11* |
|---|---|---|---|---|---|---|---|---|---|---|---|
| Exp | ∞ | 19.0 | 9.0 | 5.7 | 4.0 | 3.0 | 2.3 | 1.9 | 1.5 | 1.2 | 1.0 |
| Obs[a] | 25 | 11.8 | 5.5 | 3.8 | 2.4 | 1.9 | 1.9 | 1.8 | 1.5 | 1.1 | 1.0 |

[a]The imbalance factor is defined as the ratio of allele intensities in the tumour sample relative to the ratio of the alleles in normal DNA (Figure 1b; lane 11).

observed at the postion of the lost allele. In an attempt to simulate incomplete LOH, we performed a titration experiment by mixing genomic DNA from an individual I, being homozygous (2/2) for D3S1238, with increasing amounts of DNA from a heterozygous (1/2) individual II. Thus, the DNA from individual I mimics DNA from a tumour from individual II in which allele 1 is lost by somatic recombination. PCR amplification was performed on a total of 15 ng input DNA of each ratio for 28 cycles (Figure 1a). Thus lane 2 mimics the situation in which a tumour is contaminated with 10% normal cells. The signal for the 'contaminating' allele 1 proportionally increased with the amount of normal heterozygous DNA in the mixtures. The intensity difference between alleles 1 and 2 remain clearly visible at least up to lane 7. This would suggest that an allelic imbalance would still be detectable in a tumour with 60% non-malignant contamination. Figure 1b shows the quantitation of the alleles. Intensities of the bands in the indicated windows were scanned by laser densitometry. Imbalance factors were calculated according to the definition described in the Materials and methods section. Results obtained by this experiment are shown in Table I. As expected from the predicted values, the decrease of the imbalance factor parallels the increasing percentages of contaminating DNA, although there is no absolute correspondence between the observed and expected data.

In addition, the effect of the number of PCR cycles was investigated. This was of interest because it is known that the yield of PCR products is only proportional to the amount of input DNA template when the amplification remains within the exponential range of the PCR reaction. If not so, differences in the initial concentrations of the alleles will be compensated (Noonan *et al.*, 1990). The behaviour of the PCR products was studied by mixing DNA from two individuals, I and II, both homozygous for two different alleles of D6S251. DNA was diluted serially according to the ratios indicated in Figure 2a. The total amount of DNA in each sample was again 15 ng. Samples were amplified for 28, 33 and 36 cycles respectively. Signal intensity differences due to different ratios of input DNA are clearly visible and remain stable during increasing numbers of amplification cycles.

Figure 2b shows the quantitation of the allelic imbalance obtained after 28 cycles. There is a good agreement between the expected and observed imbalance-factors for each ratio after 28 cycles of amplification (Table II). Thus the number of cycles does not influence the outcome of the PCR reaction and does not interfere with the possibility of detecting small differences in allele concentrations.

### *Detection of LOH*

Ovarian tumours were screened with polymorphic probes in Southern analysis to select unequivocal cases of LOH. Allele losses with the RFLP markers D17S58, D17S74 and D17S4 are shown in Figure 3a. In the tumour, one of the alleles is always almost completely absent. Three cases of serous adenocarcinoma of the ovary were selected for analysis of their cognate paraffin embedded tissues on the basis of apparent loss of heterozygosity: OV16, OV26, OV29a and OV29b (a primary tumour and a metastasis). In addition OV19 and OV4 were included, the latter one to examine the possibility of detecting LOH in archival material (> 10 years of storage).

A sample of 2 µl of the extracted DNA was used as template in PCR. An estimation by dot-blot hybridisation with a BLUR8 probe (Figure 4) (not shown for OV19) indicated that these samples contained between 1 and 70 ng DNA.

The microsatellite markers 42D6, 46E6 and THRA1 were used because they are flanked by the RFLP markers D17S74 and D17S4 used in the Southern analysis (Figure 3b). To verify the results, marker 46E6 was amplified on DNA isolated from peripheral blood leucocytes and snap-frozen tissue (Figure 3c). Patients 16 and 26 are homozygous for this marker (Figure 3b). Patients 29 and 4 are heterozygous (lanes 29n and 4n) and show clear LOH: a strong intensity decrease in the tumour lanes of either the lower alleles (29a,b) or the upper bands (4a,b). Using microsatellite

**Figure 2** **a**, The behaviour of allelic imbalances during increasing numbers of PCR cycles was studied by mixing a total of 15 ng DNA from individuals I and II in the ratios 10:0 (lane 1), 9:1 (lane 2), 8:2 (lane 3), 7:3 (lane 4), 6:4 (lane 5), 5:5 (lane 6), 4:6 (lane 7), 3:7 (lane 8), 2:8 (lane 9), 1:9 (lane 10) and 0:10 (lane 11). PCR was carried out for respectively 28, 33 and 36 cycles with the marker D6S251. Exposure was overnight. At D6S251, person I is homozygous for allele 1, and person II is homozygous for allele 2. **b**, Intensities of the bands in the indicated windows after 28 cycles of amplification (right panels) were scanned by a laser densitometer (left panels).

**Table II** Expected and observed imbalance factors obtained in a titration experiment studying the behaviour of allelic imbalances

| *Lane* | *1* | *2* | *3* | *4* | *5* | *6* | *7* | *8* | *9* | *10* | *11* |
|---|---|---|---|---|---|---|---|---|---|---|---|
| Exp | ∞ | 9.0 | 4.0 | 2.3 | 1.5 | 1.0 | 0.7 | 0.4 | 0.3 | 0.1 | 0.0 |
| Obs[a] | ∞ | 8.1 | 3.4 | 1.9 | 1.3 | 1.0 | 0.6 | 0.4 | 0.3 | 0.1 | 0.0 |

[a]The imbalance factor is defined as the ratio of allele intensities in the tumour sample relative to the ratio of the alleles in normal DNA (Figure 2a; left panel lane 6).

**Figure 3** Analysis of LOH with polymorphic chromsome 17 markers in ovarian tumours. n = normal DNA; a, b = tumour DNA. Patient identification numbers on the top. **a**, Southern hybridisation with the probes D17S58 (upper panel), D17S74 (middle panel), and D17S4 (lower panel). **b**, PCR with microsatellite markers THRA1 (upper panel), 42D6 (middle panel) and 46E6 (lower panel) on DNA isolated from fixed, embedded tissue. **c**, PCR with microsatellite marker 46E6 on DNA isolated from blood and fresh (snap-frozen) tissue.

markers 42D6 and THRA1, OV19 and OV29 show loss of the upper alleles. However the loss of the upper allele of OV19 is not complete (lanes 19a,b), due to contamination with normal DNA.

We conclude that LOH can be detected in DNA isolated from fixed paraffin embedded tissue, even in DNA extracted from 10-year-old archival tissue. The observed allele losses are quite comparable to those obtained with the flanking RFLPs in Southern analysis. The very low intensity of the alleles in lane 4n is probably due to the lower amount of input DNA as verified in the dot-blot hybridisation (Figure 4).

### Discussion

Studies on selective loss of genetic material have lead to an increased understanding of the genetic mechanisms underlying the initiation and progression of a variety of human malignancies, including retinoblastoma (Cavenee *et al.*, 1983), Wilms tumour (Koufos *et al.*, 1984), familial adenomatous polyposis (Solomon *et al.*, 1987) and breast carcinoma (Devilee *et al.*, 1991). In these studies, LOH or allelic imbalance was analysed by Southern blot techniques of conventional RFLPs, requiring relatively large amounts of DNA. The major disadvantage of conventional RFLPs is the

**Figure 4** Quantitation of DNA by dot-blot hybridisation with the BLUR8 probe (overnight exposure), patient numbers and DNA source are indicated (n = isolated from normal tissue, a or b = isolated from tumour tissue).

limited heterozygosity, being maximally 50% for a two-allele marker. The introduction of the polymerase chain reaction (PCR) using thermostable DNA polymerase made it possible to use small amounts of input DNA. PCR also enables the analysis of a new class of polymorphisms based on the simple sequence tandem repeats, especially on the sequence $(dC.dA)_n.(dG.dT)_n$ (Weber & May, 1989; Smeets *et al.*, 1989; Tautz, 1989). This group of DNA polymorphisms have collectively been named microsatellites (Litt & Luty, 1989). Microsatellite markers have already proved to be very useful in mapping of several genetic disease genes (Hanzlik *et al.*, 1990; Wijmenga *et al.*, 1990; Bell *et al.*, 1991; Cannon-Albright *et al.*, 1992) and genome mapping (Jabs *et al.*, 1991; Rose, 1991). Microsatellite markers show a number of obvious advantages over Southern blot approaches as they (i) can be detected by PCR, (ii) are not restricted to telomeric or centromeric regions, (iii) are multi-allelic, (iv) can be used in a multiplex PCR (multiple primer sets per PCR reaction) and (v) need a very small amount of input DNA. Due to relatively short PCR products to be analysed, results are also obtained on partially degraded DNA, which is virtually impossible with Southern techniques.

The use of microsatellite markers in LOH studies has also recently been demonstrated by Louis *et al.* (1992) in small and archival human brain tumour specimens, using several chromosome 10 microsatellite markers. In our study we have also successfully applied microsatellite markers in the detection of loss of heterozygosity in fresh ovarian tumour samples on chromosome 17q. The results were supported by conventional Southern blotting with flanking markers. The involvement of chromosome 17 in the etiology of ovarian cancer, detected by microsatellite markers was already shown by Smith *et al.* (1992). We have modified the method in such a way that even formalin fixed, paraffin embedded sections can be investigated for LOH. We detected LOH in archival ovarian material with several microsatellite markers, even in tissue which has been fixed more than 10 years ago.

From the titration experiments in our study we conclude that LOH remains detectable despite a variable number of amplification cycles and a contamination up to 60% with normal DNA. This percentage of contamination is dependent on the genetic mechanism involved in LOH, which may lead to different allelic ratios in the tumour. Thus a monosomy would result in a 1:0 ratio, while a recombination event generates a 2:0 ratio (Cavenee *et al.*, 1983). Indeed, a 4:0 ratio may be achieved if the latter is followed by tetraploidisation, which is thought to be an important step in the tumourigenesis of breast and ovarian cancer. Obviously, the detection of a 1:0 allelic ratio is more sensitive to contamination from normal cells than a 4:0 ratio. If we assume an arbitrary imbalance factor of 1.3, LOH due to recombination can principally still be detected despite 80% contaminating DNA. In our study a somatic recombination event was simulated in which visually LOH can be detected in the presence of 60% contaminating DNA. To reduce the chance of missing LOH, samples with a minimum of 60% tumour cells should be used.

Although choice of fixative and fixation time can be critical factors influencing the outcome of PCR amplification of DNA extracted from paraffin-embedded material (Greer *et al.*, 1991*a*; Greer *et al.*, 1991*b*; Ben Ezra *et al.*, 1991), we believe that PCR-based microsatellite polymorphisms are excellent tools in the detection of loss of heterozygosity in fresh (snap-frozen) as well as in formalin fixed, paraffin embedded material. The possibility of using fixed, paraffin embedded material will be particularly valuable in opening huge pathologic archives for genetic investigation.

This work was supported by the Praeventiefonds no. 28-1666. We thank Dr Mark Skolnick for providing microsatellite primer sequences and Dr Anne-Marie Cleton-Jansen, Dr Mark van de Vijver and Andrea van Elzas for inspiring discussions.

### References

BARKER, D., WRIGHT, E., FAIN, P., GOLDGAR, D., SKOLNICK, M., LATT, S. & WILLARD, H. (1987*a*). Thirty new chromosome 17 DNA markers. *Cytogenet. Cell. Genet.*, **46**, 576.

BARKER, D., WRIGHT, E., NGUYEN, K., CANNON, L., FAIN, P., GOLDGAR, D., BISHOP, D.T., CAREY, J., BATY, B. & KIVLIN, J. (1987*b*). Gene for von Recklinghausen neurofibromatosis is in the pricentromeric region of chromosome 17. *Science*, **236**, 1100–1102.

BELL, G.I., XIANG, K.S., NEWMAN, M.V., WU, S.H., WRIGHT, L.G., FAJANS, S.S., SPIELMAN, R.S. & COX, N.J. (1991). Gene for non-insulin-dependent diabetes mellitus (maturity-onset diabetes of the young subtype) is linked to DNA polymorphism on human chromosome 20q. *Proc. Natl Acad. Sci. USA*, **88**, 1484–1488.

BEN EZRA, J., JOHNSON, D.A., ROSSI, J., COOK, N. & WU, A. (1991). Effect of fixation on the amplification of nucleic acids from paraffin-embedded material by the polymerase chain reaction. *J. Histochem. Cytochem.*, **39**, 351–354.

CANNON-ALBRIGHT, L.A., GOLDGAR, D.E., MEYER, L.J., LEWIS, C.M., ANDERSON, D.E., FOUNTAIN, J.W., HEGI, M.E., WISEMAN, R.W., PETTY, E.M., BALE, A.E., OLOPADE, O.I., DIAZ, M.O., KWIATKOWSKI, D.J., PIEPKORN, M.W., ZONE, J.J. & SKOLNICK, M.H. (1992). Assignment of a locus for familial melanoma, MLM, to chromosome 9p13–p22. *Science*, **258**, 1148–1152.

CASKEY, C.T. (1987). Disease diagnosis by recombinant DNA methods. *Science*, **236**, 1223–1229.

CAVENEE, W.K., DRYJA, T.P., PHILLIPS, R.A., BENEDICT, W.F., GODBOUT, R., GALLIE, B.L., MURPHREE, A.L., STRONG, L.C. & WHITE, R.L. (1983). Expression of recessive alleles by chromosomal mechanisms in retinoblastoma. *Nature*, **305**, 779–784.

CHURCH, G.M. & GILBERT, W. (1984). Genomic sequencing. *Proc. Natl Acad. Sci. USA*, **81**, 1991–1995.

DEININGER, P.L. & SCHMID, C.W. (1979). A study of the evolution of repeated DNA sequences in primates and the existence of a new class of repetitive sequences in primates. *J. Mol. Biol.*, **127**, 437–460.

DEVILEE, P., VAN DEN BROEK, M., KUIPERS DIJKSHOORN, N., KOLLURI, R., KHAN, P.M., PEARSON, P.L. & CORNELISSE, C.J. (1989). At least four different chromosomal regions are involved in loss of heterozygosity in human breast carcinoma. *Genomics*, **5**, 554–560.

DEVILEE, P., VAN VLIET, M., BARDOEL, A., KIEVITS, T., KUIPERS DIJKSHOORN, N., PEARSON, P.L. & CORNELISSE, C.J. (1991). Frequent imbalance of marker alleles for chromosome 1 in human primary breast carcinoma. *Cancer Res.*, **51**, 1020–1025.

FOULKES, W., BLACK, D., SOLOMON, E. & TROWSDALE, J. (1991). Allele loss on chromsome 17q in sporadic ovarian cancer. *Lancet*, **338**, 444–445.

GREER, C.E., LUND, J.K. & MANOS, M.M. (1991*a*). PCR amplification from paraffin-embedded tissues: recommendations on fixatives for long-term storage and prospective studies. *PCR Meth. & Applic.*, **1**, 46–50.

GREER, C.E., PETERSON, S.L., KIVIAT, N.B. & MANOS, M.M. (1991*b*). PCR amplification from paraffin-embedded tissues. Effects of fixative and fixation time see comments. *Am. J. Clin. Pathol.*, **95**, 117–124.

HANZLIK, A.J., BINDER, M., LAYTON, W.M., ROWE, L., LAYTON, M., TAYLOR, B.A., OSEMLAK, M.M., RICHARDS, J.E., KURNIT, D.M. & STEWART, G.D. (1990). The murine *situs inversus viscerum* (iv) gene responsible for visceral asymmetry is linked tightly to the Igh-C cluster on chromosome 12. *Genomics*, **7**, 389–393.

JABS, E.W., LI, X., COSS, C.A., TAYLOR, E.W., MEYERS, D.A. & WEBER, J.L. (1991). Mapping the Treacher Collins syndrome locus to 5q31.3-33.3. *Genomics*, **11**, 193–198.

KOUFOS, A., HANSEN, M.F., LAMPKIN, B.C., WORKMAN, M.L., COPELAND, N.G., JENKINS, N.A. & CAVENEE, W.K. (1984). Loss of alleles at loci on human chromosome 11 during genesis of Wilms' tumour. *Nature*, **309**, 170–172.

LITT, M. & LUTY, J.A. (1989). A hypervariable microsatellite revealed by *in vitro* amplification of a dinucleotide repeat within the cardiac muscle actin gene. *Am. J. Hum. Genet.*, **44**, 397–401.

LOUIS, D.N., VON DEIMLING, A. & SEIZINGER, B.R. (1992). A $(CA)_n$ dinucleotide repeat assay for evaluating loss of allelic heterozygosity in small and archival human brain tumor specimens. *Am. J. Pathol.*, **141**, 777–782.

MILLER, S.A., DYKES, D.D. & POLESKY, H.F. (1988). A simple salting out procedure for extracting DNA from human nucleated cells. *Nucleic Acids Res.*, **16**, 1215.

NOONAN, K.E., BECK, C., HOLZMAYER, T.A., SHIM, J.E., WUNDER, J.S., ANDRULIS, I.L., GAZDAR, A.F., WILLMAN, C.L., GRIFFITH, B., VON HOFF, D.D. & RONINSON, I.B. (1990). Quantitative analysis of MDR1 (multidrug resistance) gene expression in human tumors by polymerase chain reaction. *Proc. Natl Acad. Sci. USA*, **87**, 7160–7164.

PONDER, B. (1988). Cancer: Gene losses in human tumours. *Nature*, **335**, 400–402.

ROSE, E.A. (1991). Applications of the polymerase chain reaction to genome analysis. *FASEB J.*, **5**, 46–54.

SAIKI, R.K., GELFAND, D.H., STOFFEL, S., SCHARF, S.J., HIGUCHI, R., HORN, G.T., MULLIS, K.B. & ERLICH, H.A. (1988). Primer-directed enzymatic amplification of DNA with a thermostable DNA polymerase. *Science*, **239**, 487–491.

SAIKI, R.K., SCHARF, S., FALOONA, F., MULLIS, K.B., HORN, G.T., ERLICH, H.A. & ARNHEIM, N. (1985). Enzymatic amplification of beta-globin genomic sequences and restriction site analysis for diagnosis of sickle cell anemia. *Science*, **230**, 1350–1354.

SHIBATA, D.K., ARNHEIM, N. & MARTIN, W.J. (1988). Detection of human papilloma virus in paraffin-embedded tissue using the polymerase chain reaction. *J. Exp. Med.*, **167**, 225–230.

SMEETS, H.J., BRUNNER, H.G., ROPERS, H.H. & WIERINGA, B. (1989). Use of variable simple sequence motifs as genetic markers: application to study of myotonic dystrophy. *Hum. Genet.*, **83**, 245–251.

SMITH, S.A., EASTON, D.F., EVANS, D.G.R. & PONDER, B.A.J. (1992). Allele losses in the region 17q12-21 in familial breast and ovarian cancer involve the wild-type chromsome. *Nature Genet.*, **2**, 128–131.

SOLOMON, E., VOSS, R., HALL, V., BODMER, W.F., JASS, J.R., JEFFREYS, A.J., LUCIBELLO, F.C., PATEL, I. & RIDER, S.H. (1987). Chromsome 5 allele loss in human colorectal carcinomas. *Nature*, **328**, 616–619.

TAUTZ, D. (1989). Hypervariability of simple sequences as a general source for polymorphic DNA markers. *Nucleic Acids Res.*, **17**, 6463–6471.

TOGUCHIDA, J., ISHIZAKI, K., NAKAMURA, Y., SASAKI, M.S., IKENAGA, M., KATO, M., SUGIMOTO, M., KOTOURA, Y. & YAMAMURO, T. (1989). Assignment of common allele loss in osteosarcoma to the subregion 17p13. *Cancer Res.*, **49**, 6247–6251.

VOGELSTEIN, B., FEARON, E.R., HAMILTON, S.R., KERN, S.E., PREISINGER, A.C., LEPPERT, M., NAKAMURA, Y., WHITE, R., SMITS, A.M. & BOS, J.L. (1988). Genetic alterations during colorectal-tumor development. *N. Engl. J. Med.*, **319**, 525–532.

WEBER, J.L. & MAY, P.E. (1989). Abundant class of human DNA polymorphisms which can be typed using the polymerase chain reaction. *Am. J. Hum. Genet.*, **44**, 388–396.

WIJMENGA, C., FRANTS, R.R., BROUWER, O.F., MOERER, P., WEBER, J.L. & PADBERG, G.W. (1990). Location of facioscapulohumeral muscular dystrophy gene on chromosome 4. Lancet, **336**, 651–653.

WRIGHT, D.K. & MANOS, M.M. (1990). Sample preparation from paraffin-embedded tissues. In *PCR Protocols: A Guide to Methods and Applications*, Innes, M.A., Gelfand, D.H., Sninsky, J.J. & White, T.J. (eds.). pp. 153–158. Academic Press: Berkeley.