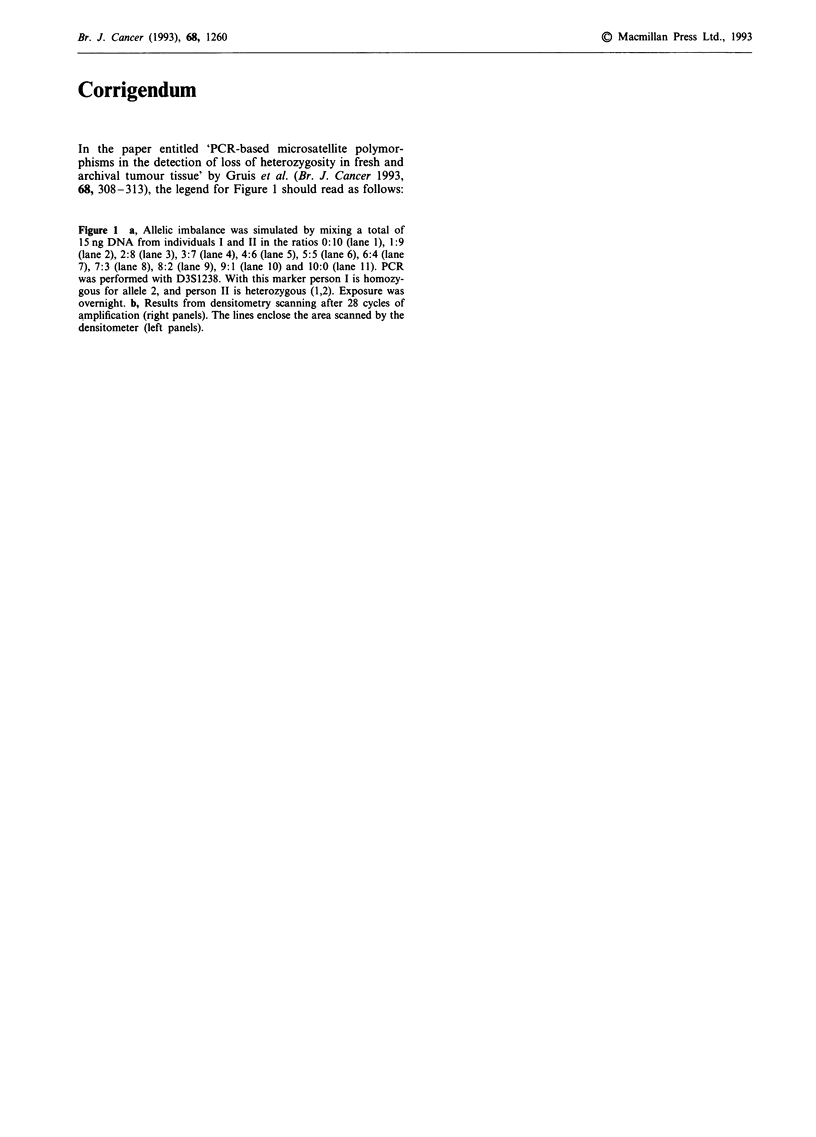# Corrigendum

**Published:** 1993-12

**Authors:** 


*Br. J. Cancer* (1993), **68**, 1260 © Macmillan Press Ltd., 1993

### Corrigendum

In the paper entitled 'PCR-based microsatellite polymorphisms in the detection of loss of heterozygosity in fresh and archival tumour tissue' by Gruis *et al.* (*Br. J. Cancer* 1993, **68**, 308–313), the legend for Figure 1 should read as follows:

**Figure 1** **a**, Allelic imbalance was simulated by mixing a total of 15 ng DNA from individuals I and II in the ratios 0:10 (lane 1), 1:9 (lane 2), 2:8 (lane 3), 3:7 (lane 4), 4:6 (lane 5), 5:5 (lane 6), 6:4 (lane 7), 7:3 (lane 8), 8:2 (lane 9), 9:1 (lane 10) and 10:0 (lane 11). PCR was performed with D3S1238. With this marker person I is homozygous for allele 2, and person II is heterozygous (1,2). Exposure was overnight. **b**, Results from densitometry scanning after 28 cycles of amplification (right panels). The lines enclose the area scanned by the densitometer (left panels).